# Cyclosporine for omalizumab-refractory chronic urticaria: a report of five cases

**DOI:** 10.1186/s13223-023-00820-4

**Published:** 2023-08-29

**Authors:** Anthony F. LaCava, Olajumoke O. Fadugba

**Affiliations:** 1 Department of Allergy and Clinical Immunology, Cleveland Clinic, 224 W. Exchange Street, Suite 380, Akron, OH 44302 USA; 2 Division of Pulmonary, Allergy, & Critical Care Medicine, Section of Allergy & Immunology, Perelman School of Medicine, University of Pennsylvania, Philadelphia, PA 19104 USA

**Keywords:** Chronic spontaneous urticaria, Refractory urticaria, Cyclosporine, Omalizumab

## Abstract

**Background:**

While both the AAAAI/ACAAI and the EAACI/GA^2^LEN/EuroGuiDerm/APAAACI guidelines recommend starting cyclosporine for patients with chronic urticaria who have had an inadequate response to omalizumab, many clinicians are hesitant to initiate cyclosporine due to paucity of clinical data. The objective of this study was to report real-life clinical outcomes in adult patients with chronic urticaria who had an inadequate response to omalizumab and were switched from omalizumab to cyclosporine. Medical records of adult patients with chronic urticaria who had an inadequate response with omalizumab and were later treated with cyclosporine were reviewed retrospectively. Data pertaining to treatment method, clinical response, and adverse effects were recorded.

**Results/presentation of cases:**

Five patients with omalizumab-refractory chronic urticaria, three of whom also had angioedema and one with an inducible urticaria, were treated with low doses of oral cyclosporine (1–3 mg/kg/d). Four of five patients in this case series had complete resolution of symptoms with oral cyclosporine, while continuing other standard therapies. Systemic side effects occurred in three patients which prompted drug discontinuation in two patients.

**Discussion:**

Cyclosporine alone was effective in inducing urticaria control in adult patients with chronic urticaria who had an inadequate response to omalizumab, though the impact of cyclosporine was limited by reversible adverse effects. Adverse effects were associated with pre-existing medical conditions. As novel chronic urticaria therapies are being investigated, this experience highlights the importance of uncovering chronic urticaria subtypes which tend to respond to cyclosporine, while providing alternative treatments with better tolerability.

## Background

Chronic spontaneous urticaria (CSU) has an estimated annual prevalence of approximately 1% of the population [1]. Up to 50% of patients with CSU remain symptomatic despite first- and second-line treatment with second-generation H1-antihistamines up to 4-times the FDA-recommended dosage [2]. For patients with antihistamine-refractory CSU, the EAACI/GA^2^LEN/EuroGuiDerm/APAAACI guidelines recommend omalizumab as step three therapy and cyclosporine as step four, while the AAAAI/ACAAI guidelines provide multiple options for step four including omalizumab, cyclosporine, other immunosuppressants and anti-inflammatory agents [3].

Omalizumab, an IgE-targeting monoclonal antibody, was FDA approved for antihistamine refractory CSU in 2014. Unfortunately approximately 30% of patients do not achieve adequate control of their symptoms with omalizumab [4, 5]. The patient subpopulation who are antihistamine resistant and respond inadequately to omalizumab represents a significant challenge for the physician. These patients may require alternative agents, and an effective option recommended by both guidelines is cyclosporine.

Cyclosporine, a calcineurin inhibitor, lacks FDA approval for CSU in the United States but can be used off-label. Clinical response rates of 40–100% have been reported with cyclosporine for CSU [6, 7]. While both the AAAAI/ACAAI and EAACI/GA^2^LEN/EuroGuiDerm/APAAACI guidelines recommend cyclosporine as an option for patients who have had an inadequate response to omalizumab, many clinicians are hesitant to initiate cyclosporine treatment due to concerns for side effects and safety. Furthermore, there is little data regarding outcomes with cyclosporine after omalizumab is failed. This case series aims to provide clinicians with real-life clinical outcomes of cyclosporine in patients with omalizumab-refractory chronic urticaria.

Baseline investigations included complete blood count, urea, creatinine, serum electrolytes, blood sugar, and urine examination. Repeat tests were performed every 4 weeks. Blood pressure was monitored at least every 2 weeks during the treatment period. Clinical response was based upon patient observation.

This study was approved by the Institutional Review Board. Table 1 summarizes the patients’ clinical characteristics and laboratory studies. Table 1 also includes the doses of omalizumab, cyclosporine, and antihistamines for each patient. Cyclosporine dosage was categorized into two groups: very low dose (< 2 mg per kilogram per day) and low dose (2–3.99 mg per kilogram per day).

**Table 1:** Clinical outcomes and tolerability with cyclosporine for cohort of patients with omalizumab-refractory chronic urticaria

	Patient #1	Patient #2	Patient #3	Patient #4	Patient #5
Age	24	35	41	39	58
Gender	Female	Male	Female	Male	Female
Comorbidities	None	Vitiligo, chronic rhinitis	Hypertension, headaches, chronic rhinitis	Asthma, chronic rhinitis, hyperthyroidism	Asthma, chronic rhinitis, thyroid cancer (s/p thyroidectomy), hypertension
BMI	26 kg/m^2^	36 kg/m^2^	35 kg/m^2^	33 kg/m^2^	34 kg/m^2^
Urticaria type	Chronic spontaneous urticaria	Chronic spontaneous urticaria	Chronic spontaneous urticaria	Chronic inducible urticaria	Chronic spontaneous urticaria
Urticaria Duration	7 months	12 months	9 months	18 months	12 months
Pertinent laboratory studies	ANA, RF, and cryoglobulin negative. Normal TSH. Anti-TPO and Anti-thyroglobulin antibodies negative	ANA negative. Normal TSH	Normal TSH	ANA negative. Anti-TPO and Anti-thyroglobulin antibodies negative	ANA negative. Anti-thyroglobulin antibodies negative
Prior use of corticosteroids	Yes	No	Yes	No	Yes
Omalizumab Dose	300 mg every 4 weeks	300 mg every 4 weeks	300 mg every 4 weeks	300 mg every 4 weeks	300 mg every 4 weeks
Omalizumab treatment duration	20 weeks	48 weeks	12 weeks	52 weeks	12 weeks
Cyclosporine Dose (milligram per kilogram per day)	3	1	1	1; dose later increased to 3	1.25; dose later increased to 3
Cyclosporine dosage category^a^	Low-Dose	Very Low-Dose	Very Low-Dose	Very Low-Dose (initially) low-dose was later used due to lack of clinical improvement	Very low-dose (initially) low-dose was later used during a recurrence when symptoms failed to respond to very low doses
Cyclosporine treatment duration	16 weeks	16 weeks	30 weeks	44 weeks	24 weeks
Concomitant medications^b^	Cetirizine 20 mg twice daily, Ranitidine 150 mg twice daily	Fexofenadine 360 mg twice daily, Ranitidine 150 mg twice daily	Cetirizine 20 mg twice daily, Ranitidine 150 mg twice daily, Hydroxyzine 25 mg daily every evening, Montelukast 10 mg daily	Cetirizine 10 mg twice daily, Fexofenadine 180 mg daily, Hydroxyzine 25 mg daily every evening, Ranitidine 150 mg twice daily, Montelukast 10 mg daily	Cetirizine 20 mg twice daily, Ranitidine 150 mg twice daily, Montelukast 10 mg daily
Clinical Response to cyclosporine^c^	Complete response	Complete response	Complete response	Near-complete improvement of cholinergic urticaria, moderate improvement of pressure-induced urticaria	Complete response
Time to clinical improvement on cyclosporine therapy	2 weeks	1 week	2 weeks	24 weeks	1 week
Relapse (if remission had occurred)	No	No	Yes	N/A	Yes
Adverse effects of cyclosporine	None	None	During second course of cyclosporine developed hypertension (did not prompt drug discontinuation)	Hypertension, hyperglycemia, hematuria, elevated uric acid (prompting cessation)^d^	During second course of cyclosporine developed elevated creatinine (peak 1.24 from baseline 1.00), prompting cessation^e^

^a^Cyclosporine dosage is categorized into 2 groups: (1) very low (< 2 mg/kg/d) and (2) low (2–3.99 mg/kg/d) dose

^b^Same doses were used during omalizumab and cyclosporine treatment

^c^Patient observation, which informed provider documentation, was used to characterize clinical response rates

^d^Blood pressure, blood glucose, serum uric acid, and hematuria normalized after cessation of cyclosporine

^e^Serum creatinine returned to baseline after cessation of cyclosporine

## Objective

The objective of this study was to report real-life clinical outcomes in adult patients with chronic urticaria who underwent treatment with omalizumab, had an inadequate response after a period of at least three months, and were switched from omalizumab to cyclosporine.

Medical records of adult patients with chronic urticaria who had an inadequate response with omalizumab and were later treated with cyclosporine were reviewed retrospectively. Data pertaining to treatment method, clinical response, and adverse effects were recorded.

## Case series

*Patient #1* A 24 year-old female with no significant past medical history presented with 7 months of hives and angioedema. She was diagnosed with chronic spontaneous urticaria. Her symptoms were refractory to maximum-dose H1- and H2-antihistamines, and omalizumab 300 mg every 4 weeks. She had been intermittently reliant upon oral corticosteroids for control of her hives. After discontinuation of omalizumab, she was started on cyclosporine 3 mg/kg/d, which resulted in complete remission of symptoms within 2 weeks. Cyclosporine was used for a total of 4 months without adverse effects. She remains in remission 6 years later and she is off all medications, including antihistamines.

*Patient #2* A 35 year-old male with a past medical history of vitiligo and chronic rhinitis presented with 1 year of hives. He was diagnosed with chronic spontaneous urticaria. His symptoms were refractory to maximum-dose H1- and H2-antihistamines, sulfasalazine, hydroxychloroquine, and omalizumab 300 mg every 4 weeks. After discontinuation of omalizumab, he was started on cyclosporine 1 mg/kg/d, which resulted in complete remission of symptoms within 1 week. Cyclosporine was used for 4 months without adverse effects. Patient was lost to follow up after hives went into remission.

*Patient #3 *A 41 year-old female with a past medical history of hypertension, headaches, and chronic rhinitis presented with 9 months of hives and angioedema. She was diagnosed with chronic spontaneous urticaria. Her symptoms were refractory to maximum-dose H1- and H2-antihistamines, montelukast, and omalizumab 300 mg every 4 weeks. She had been dependent upon prednisone 20 mg daily to control her hives. After discontinuation of omalizumab, she was started on cyclosporine 1 mg/kg/d, which resulted in complete remission of her symptoms within 2 weeks. Cyclosporine was used for 7.5 months without adverse effects. She was in clinical remission and off all medications for 2 years, after which she experienced a recurrence. H1 and H2-antihistamines were restarted and after no improvement cyclosporine 1 mg/kg/d was restarted and she again achieved remission. She developed elevated blood pressure 6 weeks after re-starting cyclosporine and was started on amlodipine. Cyclosporine was used for a total of 8 months. She remains in remission over 2 years later and she is off all medications.

*Patient #4* A 39 year-old male with a past medical history of asthma, chronic rhinitis, and hyperthyroidism presented with one and a half years of inducible hives. He was diagnosed with pressure and cholinergic urticaria based on history (provocation testing was not performed). His symptoms were refractory to maximum-dose H1- and H2-antihistamines, montelukast, sulfasalazine, hydroxychloroquine, dapsone, and omalizumab 300 mg every 4 weeks. Omalizumab was used off-label for chronic inducible urticaria. After discontinuation of omalizumab, he was initially started on cyclosporine 1 mg/kg/d. After no improvement in 6 months the cyclosporine dose was increased to 3 mg/kg/d, which resulted in near-complete improvement of cholinergic urticaria and moderate improvement of pressure-induced urticaria (improvement based upon patient observation). Cyclosporine was used for 4 additional months until he developed hypertension, hyperglycemia, hematuria, and hyperuricemia which prompted cessation. These adverse effects had developed after 44 weeks of treatment with cyclosporine. Blood pressure, blood glucose, serum uric acid, and hematuria normalized after cessation of cyclosporine.

*Patient #5* A 58 year-old female with a past medical history of asthma, chronic rhinitis, thyroid cancer (status post thyroidectomy), and hypertension presented with one year of hives and angioedema. She was diagnosed with chronic spontaneous urticaria. Her symptoms were refractory to maximum-dose H1- and H2-antihistamines, montelukast, hydroxychloroquine, dapsone, and omalizumab 300 mg every 4 weeks. She had been intermittently reliant upon oral corticosteroids for control of her hives. After discontinuation of omalizumab, she was started on cyclosporine 1.25 mg/kg/d, which resulted in complete remission of her symptoms within 1 week. Cyclosporine was used for 6 months without adverse effects. She was in clinical remission and off all medications for two years, after which she experienced a recurrence. H1- and H2-antihistamines were restarted and after no improvement cyclosporine 1.25 mg/kg/d was restarted. After minimal improvement the dose was increased to cyclosporine 3 mg/kg/d, which caused elevated creatinine approximately 4 months after cyclosporine was re-started. The creatinine elevation had prompted the drug to be discontinued. Serum creatinine returned to baseline after cyclosporine was discontinued.

## Discussion

Severe chronic urticaria refractory to omalizumab is exceedingly challenging for both the clinician and patient. Few studies have examined the clinical outcomes using cyclosporine alone in omalizumab-refractory chronic urticaria. Both Rosenblum et al [8] and Sanchez et al [9] reported that concomitant omalizumab and cyclosporine was safe and efficacious in adult patients with CSU refractory to individual immunomodulators. This case series, in contrast, describes five omalizumab-refractory patients who were switched to cyclosporine without concomitant omalizumab.

Four of five patients in this case series had complete resolution of symptoms with oral cyclosporine, while continuing other standard therapies (ie., H1 and H2 antihistamines and leukotriene receptor antagonists) according to the AAAAI/ACAAI guideline [10]. Systemic side effects occurred in three patients which prompted drug discontinuation in two patients. Increased serum creatinine was seen in one case with a rise from 1.00 mg/dL to 1.24 mg/dL. One case developed new onset hypertension and another had worsening of pre-existing hypertension which had been previously controlled with medications. The systemic adverse event frequency in this cohort was higher than previously described [7, 11]. This was unexpected considering adverse reactions occurred despite careful patient selection and the use of lower doses of cyclosporine. Two of the three patients who experienced systemic side effects had a pre-existing history of hypertension which may have served as a predisposing factor since hypertension is a known precaution for cyclosporine. These patients’ baseline hypertension was mild and the potential benefit of starting cyclosporine was deemed higher than the risk at the time of treatment. The side effects were reversible in these cases and resolved after cyclosporine was discontinued.

There are inherent limitations of this case series including the small sample size and lack of control group. The clinical course of chronic urticaria is variable and spontaneous remission may occur which was not accounted for given the nature of this study. Use of patient observation and comments to assess treatment response, rather than using validated measures of urticaria control, is also a limitation [11]. Biomarkers of urticaria were not routinely obtained on these patients. There is increasing evidence demonstrating the use of biomarkers such as baseline total IgE and basophil histamine release assay as predictors of response and nonresponse to omalizumab and cyclosporine [12]. While current AAAAI/ACAAI guidelines do not recommend routine measurement of these laboratory tests, the potential ability to predict therapeutic efficacy for antihistamine-refractory patients may propel clinicians to obtain these tests as they consider benefits and risks of therapies like cyclosporine. In addition, higher doses of omalizumab could have been attempted before considering a patient an omalizumab non-responder, and there is now a significant body of evidence to support this practice [13]. Nevertheless, this case series highlights both the beneficial aspects of cyclosporine, such as rapid and durable response, while emphasizing its potential for adverse effects, which are largely reversible.

In summary, cyclosporine was effective for five patients with omalizumab refractory chronic urticaria. In this cohort, the benefit of cyclosporine was limited by reversible adverse effects which tended to occur in patients with pre-existing medical conditions. This highlights the importance of laboratory and blood pressure monitoring during cyclosporine therapy. Further research is needed to better understand the pathophysiology of refractory chronic urticaria, to better predict the tolerability of cyclosporine, and to reliably identify predictors of response to omalizumab, cyclosporine and other therapies in the clinical setting. As novel chronic urticaria therapies are being investigated [14, 15] this experience highlights the importance of targeting chronic urticaria subtypes which tend to respond to cyclosporine, while providing alternatives with better tolerability.

## Data Availability

The authors confirm that the data supporting the findings of this study are available within the article.

## Abbreviations

CSU: Chronic spontaneous urticaria
FDA: Food and Drug Administration
IgE: Immunoglobulin E
mAb: Monoclonal antibody
mg: Milligrams
kg: Kilograms
d: Day
dl: Deciliter
